# Advances in sewage sludge application and treatment: Process integration of plasma pyrolysis and anaerobic digestion with the resource recovery

**DOI:** 10.1016/j.heliyon.2023.e19765

**Published:** 2023-09-04

**Authors:** Abdulmoseen Segun Giwa, Ndungutse Jean Maurice, Ai Luoyan, Xinxin Liu, Yang Yunlong, Zhao Hong

**Affiliations:** a School of Environment and Civil Engineering, Nanchang Institute of Science and Technology, Nanchang, 330108, China; b Institute of Environmental Science, Shanxi University, Taiyuan, 030006, China; c Jiangxi Transportation Institute Company Limited, China

**Keywords:** Anaerobic digestion, Municipal sewage sludge, Process integration, Resource utilization, Plasma pyrolysis

## Abstract

Sewage sludge (SS) is an environmental issue due to its high organic content and ability to release hazardous substances. Most of the treatments available are biological, thermal hydrolysis, mechanical (ultrasound, high pressure, and lysis), chemical with oxidation (mainly ozonation), and alkali pre-treatments. Other treatment methods include landfill, wet oxidation, composting, drying, stabilization, incineration, pyrolysis, carbonization, liquefaction, gasification, and torrefaction. Some of these SS disposal methods damage the ecosystem and underutilize the potential resource value of SS. These challenges must be overcome with an innovative technique for the improvement of SS's nutritional value, energy content, and usability. This review proposes plasma pyrolysis and anaerobic digestion (AD) as promising SS treatment technologies. Plasma pyrolysis pre-treats SS to make it digestible by AD bacteria and immobilizes the heavy metals. The addition of Char to the upstream AD process increases the quantity and quality of biogas produced while enhancing the nutrients in the digestate. These two processes are integrated at high temperatures, thus creating concerns about their energy demand. These challenges are offset by the generated energy that can run the treatment plant or be sold to the grid, generating additional cash. Plasma pyrolysis wastes can also be converted into biochar, organic fertilizer, or soil conditioner. These combined technologies' financial sustainability depends on the treatment facility's circumstances and location. Plasma pyrolysis and AD can treat SS sustainably and provide nutrients and resources. This paper explains the co-process treatment route's techno-economic prospects, challenges, and recommendations for the future application of SS valorization and resource recovery.

## 1 Introduction

Huge numbers of wastewater treatment plants generate sludge, and its production is increasing due to world population growth and industrial development. As a matter of fact, in the year 2017, about 45 million tons of municipal sewage sludge (MSS) were produced globally [1], and also after biological and chemical treatment of sewage sludge (SS), it was reported that heavy metals (HMs), organic components, phosphorous, nitrogen, pathogenic microorganisms, and other chemicals still exist. SS is a major source of secondary pollution of the environment because it contains many pollutants (polycyclic aromatic hydrocarbons, dioxins, furans, HMs, etc.). It also causes human health problems as a potential source of secondary ecological pollution, so it is very important that it is disposed of and treated properly [2]. In addition to polluting waterways with excess nutrients, SS discharges also cause fish kills and coral reef die-offs by fostering hazardous algal blooms that endanger human health. MSS is concentrated on organic matters much more than Industrial SS; the post-MSS substrate is more valuable with the potential for bio-energy, chemical, and nutrient recovery. Municipal WWTPs all over the world produce 45 million dry tons of sewage sludge annually [3]. Between 18 and 33 million tons of dry-weight SS are produced annually on average in the European Union, the United States, and China [4,5]. Given the tightening of rigorous regulatory restrictions and population growth, this number is predicted to rise. China's economic development and continuous construction of sewage drainage systems led to the high production of SS by domestic wastewater treatment. At the end of 2019, China produced approximately 39 million tons from 5476 domestic wastewater treatment plants, and 48, 23, 18, 11% were applied in landfill, incineration, land use, and construction materials, respectively [6]. MSS can be employed in biogas production and also as fertilizer owing to the presence of valuable chemical elements [7]. Therefore, after wastewater treatment, extensive, appropriate discharge and treatment of SS are crucial and highly recommended to avoid the second pollution. Due to strict discharge limits, nutrients such as phosphate recovery from SS are crucial. Maroušek et al. (2020) [8] reported the cheapest biochar that has been chemisorbed with ferric (Fe^3^) and calcium (Ca^2^). In their study, they highlighted that over 2.5 kg of P per 100 kg of modified biochar from the 80 mg P L^−1^ environment could be obtained, whereas the P forms are more readily available for plant nutrition than struvite. According to the research of Frišták et al. (2018) [9], HMs and other trace elements present in sludge inhibit its application as fertilizer in all aspects of agriculture. Other traditional SS treatments are composting, landfilling, and agricultural use.

Choosing sustainable treatment technology for MSS has to be economical, environmentally friendly, cost-effective, and socially acceptable. The worldwide industry faces numerous obstacles and dangers, including a moderate commodity market, cost-management challenges, limited access to finance, and rising expectations for social, economic, and environmental participation from host societies and regulators. Simultaneously, investors want operational rigor and capital discipline to deliver a reasonable return on assets and capital-intensive initiatives [10]. Therefore, techniques shouldn't merely solve the challenges and associated problems of SS disposal for energy gains and nutrient recovery; they should also promote a green environment by reducing greenhouse and other harmful gas emissions and, at the same time, generating economic gains. Comparing the common methods used to treat SS, each technique has its advantages and disadvantages. Researchers have looked at different ways to treat SS, such as pyrolysis [11], hydrothermal [12], incineration [13], and anaerobic digestion (AD) [14]. Thermochemical methods such as pyrolysis, incineration, carbonation, liquefaction, and gasification have attracted many researchers attention due to their sustainable energy and nutrient recovery, and also capability to reduce the toxicity of MSS [15]. Swann et al. (2017) [16] reported that the incineration process could reduce the amount of SS and get rid of organic pollutants, but it also releases furans and dioxins, and the HMs will be very concentrated in the solid ash. High amounts of syngas, hydro-oil, and biochar can be produced from hydrothermal methods such as carbonation, liquefaction, and gasification, but they consume a lot of energy. Pyrolysis has received great attention due to its ability to decompose organic matter at certain temperatures without any oxygen supply [17]. The pyrolysis process has three categories, such as slow, fast, and ultra-fast (pyrolysis); all are performed in the same way but set at different temperature levels, with <300 °C, 300–500 °C, and 500 °C, respectively. And also, the main products are syngas, oil, and char, but this process works on dry materials, and SS is reported to be highly moisturized, hence the need for the pre-treatment process.

Additionally, AD has attracted much attention for handling SS disposal because of its efficiency and can produce biogas by converting about 30–60% of organic matter, though it can take a longer start-up period of 10–30 days [18]. Because methanogenic variety and temperature sensitivity are prominent, the operating settings of AD plants frequently employ thermophilic (50–60 °C) and mesophilic (30–40 °C) conditions. The growth of AD bacteria requires certain pH ranges, whereas the optimal pH range for methanogenic growth is between 6.5 and 7.2 [19]. A suitable pH range for the hydrolysis of AD, acetogenesis, and methanogenesis is 6.0, 6.0–7.0, and 6.5–7.5, respectively. In the AD process, alkaline (pH 10) and acidic (pH 2–5) chemical pretreatments provide substantial CH_4_ yields and a high rate of organic matter decomposition in waste-activated sludge [20]. The biogas generated, after being upgraded and compressed, can be used as electricity, fuel, or for the production of heat. Despite this technique's suitability for MSS management, it has low processing capacity, is expensive, and is hard to set reaction parameters; hence, pretreatment is required. Many researchers have reported that the presence of ammonia, nitrogen, salts, and HMs in AD can inhibit the production of biogas.

Maroušek (2013) [21] wrote about the use of several pre-treatment methods, such as heat maceration, steam explosions, and pressure shockwaves, to increase the amount of biogas made from organic waste. The total methane yields (67, 179, and 255 CH_4_ VS t (^−1^)), as well as the micropore area (9, 55, and 64 m (^2^) g (^−1^)) and inhibitor formations (0, 15, and 0 mL L (^−1^)), were comprehensively examined. The pressure shock waves provided a robust treatment; however, the application of pressure shockwaves still has limitations for commercial deployment. We have examined the scientific literature on SS treatment techniques that has been published over some time. These researchers found that pyrolysis with AD [22], hydrothermal carbonization with AD [23], and pyrolysis and hydrothermal [24] are the best ways to get rid of SS. Despite these facts, there has not yet been a comprehensive assessment of SS treatment that focuses on energy recovery, bio-refinery techniques for resource recovery, nutrients and treatment scenarios with the integration of plasma pyrolysis and AD. We consider that, in all these respects, this is a timely contribution. The scale-up of both traditional and cutting-edge technologies (plasma pyrolysis and AD) may be aided by this review, which we anticipate will drive research and development activity. This review talks about a sustainable way to turn SS into energy, recover nutrients, and make bioactive compounds. It focuses on linking plasma pyrolysis with AD treatment techniques and lowering the toxicity of SS at the same time. Both processes are considered thermal technologies that operate without the use of oxygen; however, plasma pyrolysis operates at a higher temperature than the AD technique, so the coupling may be viewed as costly and energy-demanding; however, the cost can be compensated for by the quality of energy byproducts produced during operations. The various paradigm options are described, along with how individual techniques could supplement one another. Additionally, challenges, future perspectives and conclusions are presented.

## 2 General overview of sewage sludge characteristics and selected nation's scenario

### 2.1 Characteristics of municipal sewage sludge

SS is a by-product of wastewater treatment industries that contains a mixture of inorganic and organic materials. Moreover, it is generated at different stages of wastewater treatment techniques, for example; physical, chemical, biological, and nutrient removal. Besides, involving physical and chemical treatment methods in wastewater it still leads to significant amount of HMs and other recyclable organic substances. SS generated from large and developed cities is highly concentrated with metals, for example, copper, zinc, nickel, chromium, cadmium, arsenic, lead, mercury, etc., which can be harmful to human health and the environment if they are not properly managed [25]. Moreover, MSS is rich in nutrients such as nitrogen, phosphorus, and potassium, plus organic compounds such as iron, silica, and potash, which can be used as inorganic fertilizer in agriculture. The presence of nutrients and organic compounds is an essential key that allows SS to be used as a soil reformer, mostly after composting [26]. Typically, SS contains a high level of organic material, including human waste, food waste, and other organic matter that enters the wastewater system. Sludge (if not on a dry basis) is typically wet and may contain a high amount of water. Hence, properties of sludge depend on the source of wastewater, applied treatment technique, water reuse, environmental factors, treatment stage, climate change (in fact, even if SS is produced by an individual wastewater treatment plant, it has different properties each and every day), storage condition and duration, and also freezing medium [27].

**Fig. 1:**
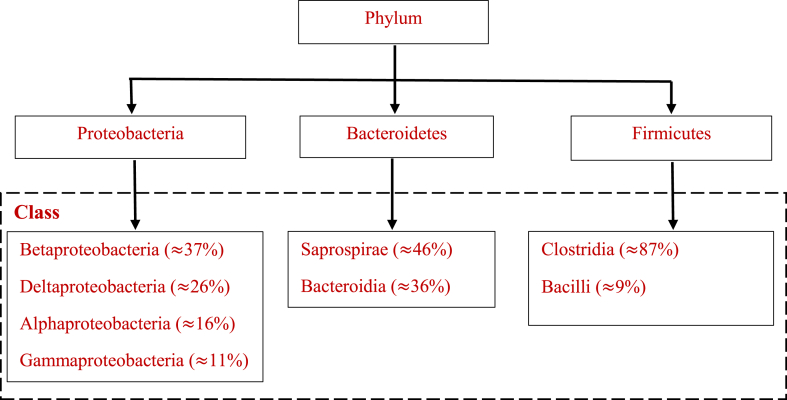
Microbial characteristics of sewage sludge [42].

Determining the suitable methods of treating SS via process integration, we found that it is important to demonstrate its physiochemical properties. Rulkens (2008) [28] grouped SS into six basic categories; (a) Around 60% non-toxic organic carbon on a dry basis, (b) phosphorous and nitrogen containing components, and (c) a large amount of inorganic and organic toxic contaminants such as heavy metals, for example, lead (Pb), zinc (Zn), mercury (Hg), cadmium (Cd), chromium (Cr), copper (Cu), arsenic (As), nickel (Ni), and other pesticides, dioxins, nonyl-phenol, polycyclic aromatic hydrocarbons, polychlorinated biphenyl sulfonates (d) Microbiological and pathogens such as protozoa, viruses, and bacteria, plus other parasites that can cause disease if they are not properly treated, for example, cestodes, nematodes, and trematodes (e) Inorganic compound, for instance aluminates, magnesium, calcium, and silicates; (f) water, that varies from a low amount up to 95% or more. Further, Table 1, Table 2, and Fig. 1 illustrate the physiochemical (nutrients), heavy metals, and the microbial characteristics of SS, respectively.

**Table 1:** Physiochemical properties of sewage sludge.

Nutrient/(mg/kg)											Reference
pH	Nitrogen	Phosphorous	Potassium	Calcium	K_2_O	P_2_O_5_	Humidity	Magnesium	Sodium	Water	
6.5 ± 0.3	39,800	20,600	1810	17,500	–	–	–	2510	320	>80%	[29]
4.3	31,800	–	11,330	12,500	1800	17,200	675,800	2200	111	–	[30]
6.72	72,800	9740	32,900	–	–	–	409,000	–	–	–	[31]
5.67 ± 0.22	52,600 ± 0.42	32,800 ± 0.26	7400 ± 0.08	17,400 ± 0.13	–	–	–	5600 ± 0.25	–	–	[32]
–	–	72,000 ± 8	8900 ± 0.7	7000 ± 2	–	2400 ± 0.7		11,000 ± 1	–	>80%	[33]

**Table 2:** Heavy metal content in sewage sludge at different regions.

Regions	Heavy Metals/(mg/kg)								Reference
	Cu	Ni	Cd	Zn	Pb	As	Cr	Hg	
Sweden	280	12	0.78	560	21	3.4	21	0.57	[34]
Turkey summer	147	75	1.7	11.33	64.2	9.4	111	0.7	[35,36]
Indıa	303	41	2.7	3085	303	63	113		[35]
China	259	56.22	10.78	906.73	81.74	28.01	188.37	4.41	[36]
USA	436	28	3.6	620	24.0	10.0	36		[37]
Thessaloniki, Greece	79	770	3.3	470	39		40		[38]
Velika Gorica, Croatia	630	109	3.06		137		74.8	2.54	[39]
Turkey winter	161	72	2.0	1299	92.8	6.5	145	0.9	[35,36]
Australia	950	64	5.3		188.0	5.6	240	40	[35]
Japan	255	40	2.3	979	53.0		69		[35]
United Kingdom	562	59	3.5	778	221.5		160		[35]
Venice, Italy	108	46	1	356	31.1	8.4	17.8		[40]
Russia	211	72	4.9	611	148.4	3.8	197	2.4	[35]

Table 1 demonstrates concentration of nutrients in SS collected from different wastewater treatments plants. Sludge exhibits slight acidic properties with pH ≈ 4.3–6.72, it is well known that pH has an effect on bioavailability of metals especially those in labile form. At very low pH value, it influence the discharge of metals, this explains that stability of metals rise as pH level increases [41]. The SS indicates significant amounts of macro-and-micro nutrients, Nitrogen, Phosphorous and Potassium, among others are considered essential elements in fertilizer. The HMs found are harmful in environment even at low concentration (Table 2). It can be seen from Table 2 that, Hg and Cd poses strong environmental risk. As presented in Table 2, different sludge present varied concentrations of HMs, this is due to the morphology of those metals in SS. Moreover, sludge is loaded with valuable inorganic nutrients which can be useful in agriculture sector.

Further studies indicates that SS has high level of bacterial as shown in Fig. 1. Original source of wastewater and biological process treatment has no tremendous effect on microbial community and their structure. The abundant bacteria phylum was proteobacteria, bacteroidetes and firmicutes in the proportion of 41.2%, 9.6% and 12.5% respectively [42]. The Presence of those bacteria in SS has positive effect in AD process, for instance proteobacteria has Beta-(37%), Delta-(26%), Alpha- (16%) and Gammaproteobacteria with 11%, and are referred as the propionate, glucose, acetate and butyrate using bacterial groups [43]. Large class in proteobacteria is identified as betaproteobacteria, as it counts around 37% of all classes of that phylum community. And also firmicutes as second largest bacterial with large class of clostridia ≈87% and bacilli ≈9% helps in volatile fatty acid breakdown in SS and AD digester, also well known as fermenters. The main class presents in the bacteroidetes phylum are saprospirae ≈46% and bacteroidia ≈36%, they are responsible for hydrolyzation process, helps in fermentation process of organic substances and produce hydrogen, carbon dioxide and organic acids throughout AD process.

### 2.2 Developing and emerging nations sewage sludge scenario

Even though data is sparse, SS from developing and emerging nations has a high parasite concentration and heavy metal content. The biggest barrier to recycling SS in developing nations is the high microbiological concentration, and heavy metal contents, which necessitates a science-based treatment decision [44]. Affordable sludge management necessitates the participation of all sectors involved in the establishment and implementation of regulations, as well as those directly involved in its generation, treatment, reuse, or disposal. Current rules have taken various approaches, primarily dependent on local situations, but significant actions are required to accomplish the sustainable development aim [45]. In underdeveloped and developing countries, SS management is a serious challenge in line with the developing of wastewater treatment plants. Moreover, demonstrating the current use of MSS in undeveloped, developing and emerging countries are complicated, due to lack, insufficient or poor sewage drainage system and also inadequate information about their facilities optimal performance; this makes it more difficult to gather information and data. Although, China and a few other countries are exempt from reporting performance data from their waste treatment facilities, The total SS generation rose sharply in China; for instance, at the end of 2021, Beijing produced 1.65 × 10^6^ tons of dry sludge [46]. In past years, China has been using sanitary landfills, buildings, incineration, and land application as general SS disposal [47]. The SS dumping reflects that China has been focusing on the wastewater treatment rather than sludge disposal and treatment [48]. investigated pollution of SS from four municipal waste water treatment plants in Nanchang city, China; in the study, they found that sludge from those treatment plants contains toxic HMs such as Zn, Cu, Cr, Pb, Ni and Cd.

For many decades, SS have been used in agriculture sector for improving soil properties, furthermore in most countries around the world, to be exact in sub-Saharan Africa, untreated or partially treated municipal wastewater are still being used in irrigation system [49]. Composting and landfill application methods of SS have generally been important ways for dealing with this type of waste in underdeveloped and rising countries. When bacteria degrade these two ways, they are known to contribute to the emission of greenhouse gases and other polluting gases such as nitrous oxide and methane. Settled sludge from sludge treatment ponds has also been used to ‘mix’ compost from solid waste, as shown in Accra, Ghana; farmers in Ghana, Mali, and Benin have been known to bribe septic truck drivers to deposit fecal matter in their fields [50]. The most critical aspect is the SS quality, as it will influence the stabilization procedure to be used. Furthermore, there is little information on the microbiological quality of SS from developing nations, owing to inadequate analytical capacity and financial resources in many situations. Despite this, some emerging and impoverished countries are making significant attempts to characterize sludge for microbial composition. Helminth is the most resistant to traditional stabilization techniques, and it is only inactivated when the treatment is performed at temperatures above 50 °C or under extreme pH values (>12 units). As a result, processes such as composting, lime stabilization, or thermophilic aerobic or anaerobic digestion should be used to reduce their densities, but their application should be based on specific criteria, and treatment of sludge with peracetic acid reduced helminth ova concentrations in raw and treated sludge from 112 to 5.5 ova/g TS, respectively [45].

In addition, according to different studies, SS from densely and urban cities in underdeveloped and developing countries are highly concentrated with toxic HMs and other contaminants [51]. Thus, SS from rural regions are loaded with organic compound and nutrients which are essential to crops such as nitrogen and phosphorus and with minimal pollutants, it can be used in the field of agriculture as growing media, soil improver, fertilizer product in landfill [29]. The SS can be valorized with some treatment methods such as pyrolysis, hydrothermal or AD for producing bio-energy and nutrient recovery. Nguyen et al. (2021) [14] showed some pre-treatment methods to advance AD of SS. The final product of pyrolysis is biochar, bio-oil and syngas, every product can be upgraded for further application. While by hydrothermal technique, SS can undergo three process such as carbonization, liquefaction and gasification [52].

### 2.3 Developed countries sewage sludge scenario

Globally, developed countries generates high amount of SS from municipal wastewater treatment plants. HMs has effect on human metabolism and environment at large [53]. Therefore, pretreatment is required to extract HMs from SS before being applied or disposed. Furthermore, Liang et al. (2021) [13] reported that incineration of SS leads to the air pollution as during combustion by emitting greenhouse gas and other dangerous gases in atmosphere. Besides, SS contain some useful organic materials such as nitrogen and phosphorus and both are essential in agriculture sector, hence sludge as good source of minerals for improving soil properties.

Japan's innovative approach to the long-term management of SS has inspired other governments to promote initiatives aimed at the recovery of energy from SS. Increased biogas production is thought to help lessen the impact of global warming by reducing emissions of greenhouse gases. Wet-air oxidation, enzymatic hydrolysis, heat-and moisture-driven hydrolysis, and thermal oxidation are techniques the EU27 has been using as alternatives for some time, and the United States has begun to embrace them as well [54]. The need of recognizing biosolids as a renewable resource has been emphasized by US stakeholders, and Australia appears to be making progress toward alternative regulation of biosolids. The use of the term “biosolids” has also gained a lot of traction in countries like New Zealand and Australia. Most of the countries analyzed in this overview are significantly reliant on imported phosphorus (P), hence this element's presence in SS has garnered their attention. Furthermore, various international policy-making agencies and wastewater stakeholders (EurEau, HELCOM, CIWEM, NBP, AWA) facilitate the connection between regulatory frameworks, market availability, and SS recovered product quality. For (SS) recovered products such as phosphorus and compost to be economically viable and practical, robust markets must first be established [55]. In developed nations, aerobic and anaerobic fermentation are currently required parts of wastewater treatment facilities, providing SS with low levels of organic matter, often considerably stable.

In European and other western developed countries are also much concerned with SS problem. In actual fact, in 2021 around 10 million tons of dry solid was generated in Europe. And also roughly 48% was used in agriculture, while 27% was incinerated [56]. According to Durdević et al. (2019) [57] publication, at the end of 2016, Croatia produced about 21,366 tons of sludge in dry basis and this amount is expected to increase up to 10^5^ tones by the year of 2024. In Ireland, Hungary, Spain, Cyprus and Finland, the mostly adopted SS treatment methods are composting and incineration, they are also highly used in Germany, Netherlands, Austria and Belgium [58]. Those treatment techniques are chosen for SS management as influenced by the scarcity of land for agriculture and population size. Moreover, Italy produce 3.4 million tons of sludge in year of 2020, and 44% was recuperated to be used in agriculture as the most potential alternatives [59]. Some of those practiced methods for treating SS are associating with environmental and soil contamination, due to high level of HMs, and air pollution by emitting some harmful gas during incineration process. Maroušek and Stávková (2021) [60] suggested a sorbent that was subsequently activated by calcium chloride (CaCl_2_), which is capable of capturing 31.8 kg P t^−1^ from sludge water that contains 52.5 mg of extractable P L^−1^, due to the fluctuation in biowaste characteristics in the application of nutrient recovery (phosphorus). The cutting-edge sorbent binds phosphorus, generally in the most beneficial forms for plant nutrition-calcium phosphate (CaP) forms (191.5 g CaP t-^1^). The issues of HM extraction, recovering priceless nutrients (nitrogen and phosphorous) for agricultural use, and improving the production of bioenergy must be addressed using further alternatives or other potential approaches.

## 3 Municipal sewage sludge treatment and the resource recovery

In terms of bioenergy and nutrient removal, MSS can undergo different treatment techniques either non-thermo or thermo processing. For instance non-thermo process are dewatering, stabilization, composting, farm land and landfilling application and also other application such as hydrothermal (gasification, carbonization and liquefaction), incinerating, torrefaction, wet oxidation, drying and pyrolysis are regarded as thermo treatments methods. But, every single technique has benefits and drawbacks as it is well discussed below.

### 3.1 Non-thermal treatment methods

#### 3.1.1 Dewatering

This treatment techniques is performed by removing water content from SS for producing thick and concentrated sludge. Wong et al. (2016) [61] described this process as crucial and suitable for SS management, because it can improve bioenergy utilization, reduce volume of sludge and help in transportation and also lessen leachate in the landfill fields. It is challenging to apply this process on SS, due to the highly hydrated colloidal structures microbial aggregates, which is present in SS properties [62]. Fenton oxidation, microwave technique, alkaline and acid pre-treatment, ultrasound treatment, advanced oxidation process, flocculation, sonication, biological methods and electrochemical pretreatment lead to the increase of SS dewaterability [63,64]. Changing of bound water into free water, sludge characteristics can improve this process to attain high sludge solid amount. The difference between dewatering and thickening is that the latter removes substantially more water from the SS to generate a concentrated sludge product with a concentration of sludge dewatering, often between 15% and 25%. The former removes free water, raising the dry solid content to 4–6%, and the execution of the technique process is where the second difference lies [65].

Applying SS dewatering process is highly challenging and costly stage of treatment process but also it is sustainable methods as it uses organic chemicals for example flocculants. This floc is composed by microbial aggregates and their extracellular polymeric substances (EPS), which appeared in different layers such as loosely bound, tightly bound and slime. The EPS are in charge of the functional integrity, structural, porosity, specific surface area, and filtration resistance of the aggregates, and also unlike other treatment technique such as incineration, this does not contribute to the carbon emission [66]. Additionally, in China, SS dewatering and landfill was couple together as new routes for sludge disposal and this treatment was common and popular performed in many years ago [67]. In fact, this treatment techniques are associated with several environmental issues for instance emission of greenhouse gases such as methane, nitrous oxide and carbon dioxide and also it may cause the second pollution when landfilled. Even though direct landfill is economic, but for reduction of carbon discharge in ecosystem, this process it can be slowly abandoned for green environment in future, and at some point, of stage it can be taken as emergency method.

#### 3.1.2 Composting

Composting is considered as natural and biological degradation, which is performed under aerobic environment to allow the increase of thermophilic temperature. Composting process servers as source of micro and macronutrients such as nitrogen, sulphur, phosphorous and potassium, and this can be obtained from bio-wastes materials, and SS which is regarded as rich in organic material and other trace compounds [26]. In many decades ago, this method has been performed as management of organic waste substances. Therefore, this is essential technique for the SS treatment, and improving soil properties or to be good source of fertilizer in agriculture sector. However, MSS is reported to contain pathogens, this problem can be solved by composting method under thermophilic condition that destroys pathogens [68]. Moisture, temperature, pH, nutrient concentration, and oxygen availability are the primary variables that influence the biology of composting. The ideal moisture content is between 50 and 60%; moisture levels below 40% may slow down decomposition, while moisture levels above 60% will prevent substrates from achieving sufficient structural integrity. Although most mesophiles are destroyed when the temperature hits 70 °C, temperature rise is crucial for killing infections; for the majority of bacteria, the ideal pH range is between 6 and 7.5, while for fungus, it is between 5.5 and 8 [65]. Throughout the several stages of composting, the pH changes and is virtually self-regulating. Microorganisms need carbon and nitrogen as energy sources in order to grow. The composting mixture's ideal carbon to nitrogen ratio, measured in weight, should be 25 to 35. Between 5% and 15% of the gas mass in the composting mass should be kept as oxygen concentrations, and they should be evenly distributed. The process's required temperature profile will be compromised by excessive aeration.

In China, composting have been preferred and commonly recommended for treating SS. and its compost is considered as rich in organic substances and nutrients to be used as soil conditioner but high concentration of HMs limited its application. Feng et al. (2015) [69] proposed that, wastewater should be treated differently according to their original source, but most residential cities has no separate municipal wastewater discharge routes. Recently, many studies has been carried out with focus of advancing MSS disposal [70], and the development of composting approach using microorganisms and earthworms for accelerating decomposition process of organic wastes was conducted [71]. The microorganism ingest, grind and digest organic materials from waste, which will modify sludge biologically, chemically and physically. In addition, earthworms excrete vermicast and this is known to be microbial active, stable, humified and fine. Furthermore, the SS will be concentrated with valuable inorganic nutrients such as N, P and K for agriculture [72,73]. Nevertheless, composting of SS is associated with some environmental challenge such as emission of greenhouse gases for instance methane, carbon dioxide and nitrous oxide gas that contribute to global warming [74] and also this process can't eliminate toxic HMs which are highly present in SS.

#### 3.1.3 Stabilization

Stabilization refers to a process of reducing the pathogenic content and the potential for odors or other negative impacts of the sludge. There are several methods for stabilizing SS, including: Aerobic digestion, which is considered as a biological process that uses oxygen to decompose the organic matter in the sludge. This process can reduce the pathogens in the sludge, and it produces carbon dioxide and water as byproducts [75]. AD is biological process that breaks down organic matter in the absence of oxygen [14]. This process produces biogas, which is a mixture of methane and carbon dioxide, and a solid residue known as digestate. The produced biogas can be used as a fuel, while the digestate can be used as inorganic fertilizer. Few drawbacks are associated with the AD, it requires that operational parameters need to be tightly controlled in order to achieve optimum performance: Anaerobic bacteria tend to be slower growing and more sensitive to changes in conditions, and AD Inhibition with high ammonia nitrogen concentrations [76].

Kulikowska et al. (2021) [26] reported that composting can be used as method of SS stabilization. It is a natural process that uses microorganisms to break down the organic matter in the sludge. This method requires aeration and the right mix of carbon and nitrogen sources to promote decomposition. The end product of composting is a stable, soil-like material that can be used as a fertilizer or soil conditioner [77]. Thermal treatment involves subjecting the sludge to high temperatures to kill pathogens and reduce the volume of the sludge. This process can produce byproducts such as syngas and bio-oil, which can be used as a fuel, and also solid char, which can be used as additive or as soil property improver. Each of these methods has its advantages and disadvantages, depending on the specific needs and goals of the sludge treatment. In practice, different stabilization methods may be combined to achieve optimal results, depending on the characteristics of the sludge and the desired final use of the product.

However, stabilization process can be destabilized with physical, biological and chemical stressors such as temperature and time, irradiation, desiccation, pressure, cavitation, ultrasound, anaerobic mesophilic digestion, thermophilic and mesophilic aerobic digestion, pH, composting (static aerated pile, within-vessel and windrow), autothermal thermophilic aerobic digestion, oxidants and non-charged chemical. All of those stressors can destroy cell membrane, denaturate DNA or other severe cellular structure and solubilize organics. Tomei et al. (2016) [78] proposed new technique to advance sludge stabilization and improve its quality to be applied in agriculture by the separation of thick primary and the secondary sludge post aerobic digestion, and after AD. The proposed routes seems to treat SS disposal, increase sludge stabilization, and affordable with minimal environment effect. Some methods which can be performed for improving SS stabilization includes AD (degradation of organic substance in oxygen starving condition for methane, carbon dioxide production and digestate), simultaneous aerobic stabilization, alkaline stabilization (by increasing pH create unsuitable condition and inhibit growth of microorganisms in sludge), pasteurization (to kill pathogens) and also (thickening, dewatering, drying) of SS [79,80].

#### 3.1.4 Farm land treatment

SS, also known as biosolids, can be used as a soil amendment on agricultural land. Its application to the farm land can provide several benefits, including soil fertility improvement, increasing crop yields, and reducing the need for synthetic fertilizers. In fact, SS is rich in nutrients such as nitrogen, phosphorus, and potassium, which are essential for plant growth. Around 200 kg of dry sludge contains 8 kg of phosphorus, 6 kg of nitrogen and 10 kg of other essential soluble salts [81]. Moreover, sludge can help in pH modulation and provides some water content for the crop's germination. When applied to soil, these nutrients become available to plants over time, providing a slow-release source of fertilization. In addition, the organic matter in SS can improve soil structure, water-holding capacity, and nutrient retention.

However, for thousands of years, farm land treatment comes as most common methods used in China for treating SS but this methods has been reduced from 60.9% up to 21.9% in the year 2009–2017 [6]. Clearly, sharp reduction of this disposal technique show that SS have some characteristics which leads it to be un-suitable with respect to agriculture sector and some of that factor includes presence of HMs in sludge. Sludge has some phytochemical toxicity of HMs, increase in sequence Cd, Hg, As, Ni, Pb, Cr, Cu and Zn [82]. Hence the limitation of applying this process within long period of time, due to the fact that, toxic HMs can be accumulated in soil and adsorbed by plant and get to human and other living creatures. It is highly suggested that, before applying SS to agricultural land, it needs to be treated and stabilized to reduce the potential risks associated with pathogens, HMs and other contaminants. The level of treatment depends on the intended use of the sludge and the regulatory requirements in the local area. Once treated and stabilized, the SS can be applied to farm land using a variety of methods, such as surface spreading, injection, or incorporation into the soil. The application rate and frequency of SS application depend on factors such as soil type, crop type, and nutrient requirements. It is essential to note that the use of SS on agricultural land is subject to regulations and guidelines to ensure that the application is safe for human health and the environment. The appropriate management of SS as a soil amendment on agricultural land can provide a sustainable solution for both waste management and agriculture.

#### 3.1.5 Landfilling

World widely, landfilling of waste comes as second treatment method to incineration as traditional way of disposal [83]. In china, from the year of 2008–2018, wastewater treatment plants was highly constructed and advanced, this increases the total treatment capacity of municipal waste water, though it shows high amount of SS generated as inevitable product from wastewater treatment industries [84]. Currently, east china as the most developed has the highest number of wastewater treatment plants, around 28% are located in that respective region with 68.4 million m³/d treatment capacity of municipal wastewater and with associate to the increase of daily amount of wastewater treated, thus lead to the generation of 35.4 million tons (moisture based) by the end of 2018 [85]. However, because of improper disposal of SS; nowadays landfilling is among dominant method used to treat this kind of waste product in the northeast China (excluding Beijing) and also particularly Handan, landfilling rate of SS is up to 96%, and other remaining percentage is not efficiently treated. In addition, in 2016, in Shanghai, Jinhua, Wenzhou, and Fuzhou SS landfilling was (76.3, 96.2, 84.4, 78.6) % respectively [86].

This treatment method has shortcomings, even if buried in advanced and well-designed landfill; still it is not ecological friendly. Owing that SS contains toxic HMs, pathogens and other organic micro pollutants, hence environmental contamination. Landfill leachate and SS both contains complex toxic substances with random and unknown concentration, when discharged into the environment, it can harm living creatures and affect working process of ecosystem [87]. Apart from this, when sludge is landfilled, it generates some greenhouse gases during decomposition of organic material. Not merely that, it leads to the waste of valuable organic substances in sludge such as nitrogen and phosphorous which can be used as good source of fertilizer. Based on environmental protection and conservation of resources, this method has to be replaced with a new and advanced alternative for SS positive use. Many countries in Europe now consider SS landfilling illegal; they are closing up such facilities, drifting gradually away from such disposal techniques, while some have advanced the capability of landfill technology towards sustainable resource recovery according to Landfill Directive 99/31/EC [88].

#### 3.1.6 Anaerobic digestion

AD is another technology that is used to treat SS, and gaining global attention [89]. Based on Giwa et al. (2020) [90] investigation on AD, this process involves the use of bacteria to break down organic material in the absence of oxygen, which leads to the production of biogas, that can be used as a source of energy, and a nutrient-rich residue/digestate which can be used as an inorganic fertilizer. Since 1900s, this process have been used for SS and bio-wastes treatment as it shows efficiency for converting around (40–60) % of biodegradable organic materials into (60–65) % of methane, (30–35)% of carbon dioxide and other traces of nitrogen, hydrogen, hydrogen sulfide plus water. For instance Albert Lea facility in Minnesota use 16.5 million gallons/day of SS to produce 75,000 ft^3^/day of biogas and also Italy uses 380 m^3^/d of SS to generate around 6100 m^3^/d of biogas [91]. Dong et al. (2018) [92] investigated the amount of SS and treatment techniques towards this waste. The current needs of modern waste water resource recovery facility approaches have encouraged the scientific community to search for a wide range of technological solutions that have allowed for more circular resource flows, which aim at resource recovery (e.g., water, nutrients, energy, and biomaterials) from wastewater, of which AD is one. This shift to a circular economic paradigm encourages a reduce-reuse-recycle strategy for SS and trash management, which contributes to the 2030 Agenda for Sustainable Development [93]. Aerobic and anaerobic fermentation are already mandatory components of wastewater treatment plants in developed countries, offering SS with low levels of organic matter (usually very stable).

Some studies has shown AD process to minimize HMs bioavailability into more stable form which is difficult to leak into the ecosystem. Moreover, digestate from this process is well known to contain high amount of valuable nutrients such as nitrogen, phosphorous, potassium and other trace organic carbon materials which can be used as source of nutrients for growing crops. Protein degradation in SS is nitrogen source. Massé et al. (2007) [94] conducted AD process on swine manure at laboratory scale, the result shows the reduction of soluble chemical oxygen demand and total solids and also almost 100% of nitrogen was recovered at the end of the process. In addition, AD process has four significant biochemical stages which are hydrolysis, acidogenesis, acetogenesis and methanogenesis. First stage of the process is the long reaction where protein, complex sugars, starches and other organic polymers are decomposed into monosaccharides and other soluble substances. Some species of bacteria included in this stage of reaction are Bacteroides, Erwinia, Ruminococcus, Clostridium, Firmicutes, Microbispora, Cellulomonas, Fibrobacter, Prevotella, Acetovibrio species, among others [95]. Furthermore, during the second and third stage, different kind of bacteria species will decompose product from the first stage into ammonia, carbon dioxide, volatile fatty acid and hydrogen sulfide. Other bacteria species such as Syntrophomonas, Syntrophobacter, Syntrophothermus, Syntrophobacter, Pelotomaculum and Syntrophus takes place in the third stage by breaking down organic acids into hydrogen, carbon dioxide and acetic acid. At the last process stage, Methanosarcina and Methanosaeta convert water and acetate into methane gas. On the other hand, Methanoculleus and Methanobacterium bacteria species can facilitate the reaction of carbon dioxide with hydrogen to produce methane gas via hydrogenotrophic methanogenesis.

Several researchers proved that high yield of methane gas can be captured in the first 15 days of the process, hence the resident time of AD is around 10–20 days [96]. Therefore, AD has outstanding performance in the production of energy, volume and pathogen reduction, heavy metals stabilization, and valuable nutrient and carbon recovery via biogas production.

**Fig. 2:**
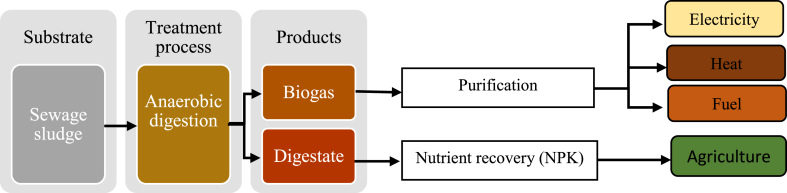
Anaerobic digestion approach of municipal sewage sludge.

Fig. 2 shows the routes of SS management through AD process to generate essential byproducts.

SS is reported to generate high amount of biogas through AD treatment method, due to the fact that it is highly loaded with organic substances. At the end of the treatment process, AD produces digestate, which can be used to amend soil properties and serve as a great source of organic fertilizer in agriculture application; since it contains precious nutrient including nitrogen, phosphorous, potassium and other organic materials. In addition, biogas can be upgraded into decent source of bio-fuel, heat and electricity. Even though this treatment method can be assumed to be suitable to handle SS disposal, Nguyen et al. (2019) [97] investigated the effect of HMs in AD process by using contaminated samples with Cu, Zn, Cr, Pb (concentrations range was between 19 and 80 ppm) separately. Thus, the result showed the decrease in biogas production about 36.33%, 31.64%, 31.64%, and 30.60% for Cu (II), Zn (II), Cr (VI), and Pb (II) compared to the control (at the concentration of 80 ppm) respectively. This imply that as the toxic HMs concentration increases in the sample, it leads to more inhibition which cause the less biogas production, furthermore at the end of this study it is clear that Cu (II) had more inhibition influence compared to the other toxic HMs in sludge. In addition, this toxic HMs can also be found in the AD digestate [98]. Hence, the waste of useful chemicals and HMs contamination at any field of this digestate may be applied. Therefore, this process must be coupled with another economic and environmentally friendly treatment technology for increasing and improving yield of biogas produced by preventing some inhibitor for instance HMs, ammonia nitrogen etc., in the process and reduce digestate toxicity level [99].

### 3.2 Thermal treatment methods

#### 3.2.1 Gasification of sludge

Hydrothermal gasification (HTG) is thermal process which is performed under temperature range above 374 °C and pressure of 22.1 MPa to convert organic substances into syngas as major product, the rate of reaction is influenced by the fall of static dielectric, density and ion dissociation [100]. In recent decades, this process has gained attention for chemical and fuels recovery from high water content organic materials. Additionally, syngas produced from this techniques is reported to contain high amount of hydrogen gas (H_2_) [101], it can be used as numerous application, for example, desulfurization of thick oil for ecological purpose, ammonium and petroleum production. However its production yield can be affected by different factors such as organic raw material, reaction time and temperature.

Presently, other alternative ways of producing H_2_ includes stream reforming of methanol, natural gas, electrolysis process and heavy oil by stream catalytic conversion, but each and every method has setback such as high energy consumption, expensive, some found unsustainable and use nonrenewable (fuels) as the raw material, hence emission of greenhouse gases [102]. When increasing temperature, H_2_ concentration increases and methane gas and carbon dioxide decreases, while by increasing sludge the amount of methane increased and H_2_ in syngas decreased, contrary by the increase of water leads to the production of H_2_ than methane gas [103]. Hence, generation of methane gas and H_2_ in syngas is thermodynamically reverse. However, HTG technique produce high amount of syngas, it also generate less hydrochar and hydro-oil [104], therefore, residues of this process has some quantities of HMs, owing to the fact that SS is highly concentrated with toxic HMs. Since HTG is applied at a high temperature rate, it means that some HMs might be present in the syngas [105,106]. It is well known that HMs in gaseous state is very reactive.

#### 3.2.2 Carbonization of sludge

This technology is well known as hydrothermal carbonization (HTC) process, which turns organic substances into high carbon material (hydro-char) and small amount of hydro-oil and syngas at certain level of temperature and pressure within particular period of time. HTC gained more attention as it accelerate SS dewaterability, hydrophobic, produce high yield of biochar (which can be used in agriculture as organic fertilizer, pH moderator and reduce inhibitor such as ammonia nitrogen in AD) and other functional materials [107]. Besides, carbon level depends on the raw organic substance and hydrothermal parameters, for instance, temperature and time. This process was economical as it consumes less energy and doesn't require pretreatment. In addition, hydro-char can be applied as solid fuel to replace fossil fuels, where during combustion of it, volatile nitrogen can be produced in form of ammonia (NH_3_) which can be upgraded to react with NO, hence the reduction of NOx emission, if it used as solid energy source [108,109]. Nevertheless, HTC is ineffective to remove HM in SS for instance, Liu et al. (2018) [110] showed hydro-char as HMs transmitter in the environment, the proposed they proposed co-process with pyrolysis as new approach for toxic HMs immobilization. Lang et al. (2019) [111] investigated the availability of toxic HMs in swine manure after HTC, the results shows that high level of HMs was captured in hydro-char from raw swine manure. This study recommended Calcium oxide to be used as additive to HTC treatment method to reduce amount of HMs in hydro-char (about 15%). By changing to stable fraction, and therefore potential environmental contamination can be reduced.

#### 3.2.3 Liquefaction of sludge

Thermochemical technique for hydrolyzing organic substance by means of water into hydro-oil is called hydrothermal liquefaction (HTL), this treatment method is operated under high temperature range of (280°C-370 °C) and high pressure of (10–25 MPa) [112]. In fact, under these parameters water (in liquid fluid) has remarkable properties for example low dielectric constant, which influence solubility with organic materials [113]. Differ from other hydrothermal methods, HTL use water as medium of reaction and catalyst. Additional benefit of this process, it generates high amount of hydro-oil without any prior treatment. Hydro-oil is produced through five different stages such as hydrolysis, dehydroxylation, decarbonation, polymerization, and aromatization. Like other treatment technology, it also recover nutrient such as phosphorous and nitrogen. However, several studies conducted towards SS reported transformation polycyclic aromatic hydrocarbons (PAHs) properties during HTL [114]. The result showed that, total amount of PAHs in hydro-oil reach 55.0–106.6 mg/kg, with high level of toxic equivalent quantity of 6.1–8.6 mg/kg. By increasing temperature and resident time, leads to the capture of PAHs into hydro-oil, while the rise of solid-liquid ratio promotes PAHs to be captured in hydro-char. The same implies to the HMs in hydro-oil, Cd was combined with organics and transferred into hydro-oil at the end of HTL process, thus impeding the use of hydro-oil in further application process [115].

#### 3.2.4 Incineration

Recently, incineration of SS was the most common performed and globally accepted as treatment route. Nowadays, large MSS incinerators are still in operation for energy generation and turning SS into ash, and also this is an oxidative process, which turns nitrogen, organic carbon, phosphorus and sulphur into gaseous [13]. Based on large (volume and pathogen) reduction, odor minimization and production of energy, in China it becomes the best alternative technology of SS disposal. Zhang et al. (2021) [116] has summarized SS generation in 31 provinces of China, Shandong and Guangdong are the top two provinces in treatment capacity, thus treating 15.62 and 23.67 million m^3^/day, individually. Moreover, in other countries, such as France, Denmark, Germany, Belgium, Japan and USA about 20%, 24%, 14%, 15%, 55% and 25% of their total produced sludge is being treated by incineration, respectively [117].

However, this technique isn't the best disposal technique of SS because about 30% remain as ash [118]. In most of the cases, this ash is dumped into the landfill, and also it is considered as harmful to the surrounding due to the high content of toxic HMs, this leads to leachability and contaminate the ground water. Besides, other limitation use of this method is gases emission pollutant, such as, non-methane volatile organic materials, nitrogen oxides and sulphur dioxide. Incineration of SS leads to the emission of ammonia [119]. Ammonia was a valuable nutrient for growing crops but also contribute to nitrogen pollution by acidification and eutrophication [120]. Whenever acid deposition increases, and lakes, ocean and soil becomes supersaturated with nutrients which harms marine ecosystem deforestation and denaturation of plant [121]. Therefore, incineration treatment method was found unsuitable for SS treatment. Recently, there have been advanced engineered incinerators with high air pollution abatement competence and resource recovery capability. It is critical to subject these technologies to economic and societal scrutiny, especially given their impact on air pollution and global climate change. A techno-economic study of short-term anthropogenic emissions of air pollutants and particulate matter, as well as their impact on social well-being and the climate, revealed comprehensive analyses of air pollutants such as PM_2.5_, PM_10_, NO_x_, SO_2_, CO, O_3_, and NH_3_, with significant improvements in air quality indicators, particularly in India and China [122].

#### 3.2.5 Plasma pyrolysis

Plasma pyrolysis is a technology that uses high-energy plasma to break down organic material into simpler molecules in absence of oxygen [123]. Also this process can be used to treat a wide range of waste materials, including SS. The benefits of plasma pyrolysis are that it can convert organic material into energy-rich gases and liquids, which can be used for energy and electricity generation, and also it can also convert organic material into a solid residue that is safe for disposal. This treatment process require high amount of temperature, where around (1700–9700) ^o^C of plasma torch to convert waste substances into small molecules. Additional benefit of plasma is to destroy toxic materials, lower generation of polluting gases such as furans and dioxins (just negligible amount), reduce waste volume up to 99% [124]. Based on plasma chemistry, residence time of complete process can vary in between milliseconds to achieve plasma arc, but depends on the raw waste material with major products of syngas and non-leachate residue. Moreover, numerous reaction occurs such as rearrangement, oxidation, elimination, substitution, addition or reduction during pyrolysis. In year of 1990, plasma pyrolysis gained much interest, as it can recover energy and other different chemicals which are present in raw waste material. Within different countries around the world for example USA has accepted plasma pyrolysis as new technology to replace incineration treatment [125]. Recently, extensive studies has been carried out toward this plasma technology, by using different kind of waste products for instance plastics, medical waste and other hazardous municipal wastes [126]. Hence, this process has considered as one of the leading in waste disposal as it can degrade waste material in economic, ecological and safe manner.

**Fig. 3:**
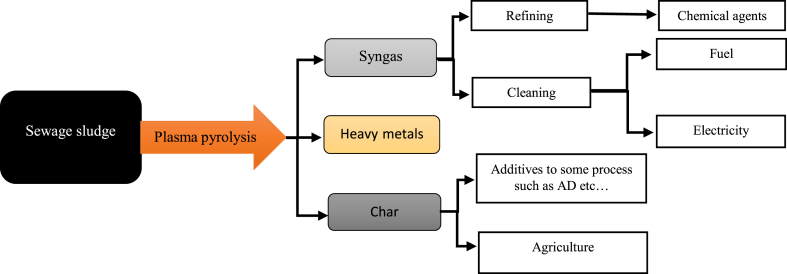
Plasma pyrolysis routes of municipal sewage sludge for bio-refinery.

In addition, during plasma pyrolysis, the plasma torch does not only act as heat supply, it also produce ultra-violet radiation and ionic radicals to accelerate the oxidation reaction. Besides, main products generated by this process is syngas which consist hydrogen, carbon monoxide, methane, carbon dioxide plus other trace gases and Char which contain high concentration of inorganic materials. Some studies was conducted to produce high amount of hydrogen from wastes, owing to its eco-friendly properties and can be used in many industrial operation. In that context, comparing plasma gasification and plasma pyrolysis, outstanding performance was shown by plasma pyrolysis in energy production; the production of H_2_ from plasma gasification is determined between (25–48) mol%, while its generation from plasma pyrolysis is calculated to be around (53–82.5) mol% [123]. Furthermore, due to the high temperature used in the process, the volatilization of HMs can be collected in gaseous state. This shows the possibility of recovering some condensable metals. Szente et al. (1998) [127] reported laboratory scale zinc recovery in oxide and metallic form, and Ye et al. (2022) [128] reported that, pyrolysis byproduct can adsorb HMs. This study investigated the performance of the cattle manure and cherry wood biochar towards toxic HMs adsorption such as Lead (Pb), Nickel (Ni) and Cadmium (Cd), the result shows the highest adsorption capacity achieved was around (40.8, 25.1 and 24.2) mg·g^−1^ respectively. Through biochar magnetization, by using Iron (II, III) oxide modified with humic acid salt can lead to the high adsorption of Mercury (Hg) in aqua environment [129]. However, this treatment process is associated with some challenges such as costly investment and system maintenance, moderation of temperature and handling syngas with extremely high temperature [123]. Beneficial and marketable byproducts from this treatment process can be a great source of income to subdue its operational cost. Additionally, Fig. 3 presents an approach of SS via plasma pyrolysis with byproducts.

This treatment process takes place under high temperature and pressure without any oxygen supply for converting organic and inorganic materials into syngas and Char with other trace hydrocarbon substances. Moreover, some chemicals such as H_2_ can be extracted from syngas, which can be upgraded to generate electricity and fuel energy [130]. Unlike other treatment process, plasma pyrolysis is environments friendly and it can be employed for treating toxic substances as all phases of matter takes place, it can influence the adsorption of HMs in gaseous state. HM has specific temperature for volatilization, while Mercury (Hg), Arsenic (As), Tin (Sn) evaporate at low temperature comparing with Lead (Pb), Zinc (Zn) and Cadmium (Cd). In addition, plasma pyrolysed residue (Char) can be used as additive in AD process to enhance production of biogas. Char can positively affect AD treatment process, by improving production of biogas in quantity/quality and promote the degradation of chemical oxygen demand [131]. Moreover, this study also discovers that digestate with Char are rich in valuable nutrients such as phosphorus, nitrogen and potassium, hence its can be used as soil conditioner or as organic fertilizer and also it shows outstanding thermal stability.

#### 3.2.6 Torrefaction

Torrefaction is sometimes defined as partial pyrolysis or roasting, as performed under temperature range of >300 °C and monitored from 15 to 60 min [132]. The optimum temperature and operation time was well discussed by Ref. [133]. In addition, this treatment technique generate large amount of bio-char, but its properties depend on torrefaction parameters such as temperature, resident time and raw biomass. Torrefaction has several remarkable advantages such as improving fuel properties, increase in energy density, reduction of oxygen/carbon which influence gasification, it takes short period of time, low energy consumption, enhance carbon fixation and hydrophobicity of torrefied biomass (than raw biomass) [134].

Moreover, Białowiec et al. (2020) [135] conducted research on toxicity of the bio-char from torrefaction of SS, the results showed that increasing temperature and resident time leads to the toxicity reduction. However, toxic HMs was independent on time and temperature. Furthermore, biochar with negligible amount of HMs can be polished before application as a soil conditioner or as fertilizer, but raw SS with high quantity of toxic HMs is not recommended to be used as an organic fertilizer. As in the same study, after the operation, Cd leachability was increased (compared to untreated), and the content was extremely high from 4.4 to 12.3 mg Cd·kg^−1^ d m (as present in raw SS), hence its exclusion to be used in agriculture. This shows that torrefaction method cannot put HMs out of the equation. Hence torrefaction is not sustainable technique for treating such waste (SS) which is loaded with high amount of toxic HMs.

#### 3.2.7 Drying

Currently, finding economic, recycle, environmental friendly and proper management of SS has been major challenges for researchers. Solar drying has been commonly applied across the world as sludge treatment, due to it is natural, affordable, and ability to destroy pathogens such as helminth egg [136]. It reduces sludge volume and serves as a source of renewable energy. Bok et al. (2017) [137] constructed hybrid greenhouses of SS in the last decade. Kamil Salihoglu et al. (2007) [138] showed operational capacity of the drying industry in Bursa city, located in the southeast of the Marmara Sea, in northwestern Turkey, about 27,000 tons of sludge (dry solid) was produced in the year of 2006. Close drying system showed great performance that open system, by reducing 40% of the sludge volume, reduce pathogen in 10 days (summer period) and changes in the dry solids content. Thus, using solar energy in sludge drying process can be an alternative way for cost reduction of drying practice. However, this process has some limitation, for instance, it is climate dependent, unstable temperature conditions, it cover large area and high chance of odor and emission of harmful gases during the process. This process can't handle the challenge of HMs which are contained in SS. Therefore after drying application further advanced technology is highly recommended to avoid second pollution.

#### 3.2.8 Pyrolysis

Recently, pyrolysis process gains much attention, due to its environmental friendly and ability to convert bio-wastes into three major products such as bio-oil, bio-char and syngas. This is a thermal process which is operated in the absence of oxygen. All pyrolysis product found to be useful and employed in many applications. Bio-char can improve production of biogas (when used as additive in AD) [139]. However, AD can be inhibited with ammonia nitrogen, sulfide, limonene, volatile fat acid, long-chain fatty acids, and pH. Biochar can help in modulation of this inhibitors for stabilizing operation in anaerobic digester, hence high production of biogas as major product of this process. It can ameliorate soil properties, as this product contain most valuable compound which is beneficial for plants. Bio-oil is a dark brown flowing fluid that contains about 400 organic complex mixture compound, roughly (75–80)wt%, (20–25)wt% of organics and water, respectively [140]. Mostly, it is considered as renewable resource for the energy production, oxygen-containing chemicals and biofuels. Besides, advanced treatment technology such as catalytic and thermal methods is recommended owing to its variety complex mixtures of organic compounds. Generally, qualitative and quantitative analysis of pyrolysis bio-oil and wide application of this valuable product can be found in Ref. [141] study.

In addition, syngas which is hydrogen, carbon monoxide, methane, carbon dioxide, water vapors and other trace hydrocarbons containing material. It is mostly applied in electricity, heat generation and also considered as good source of hydrogen [130]. However, pyrolysis process seems to be best technique for sludge treatment, this method has some limitation when it comes to the HMs containing substances. Pyrolysis of SS was a new treatment route adopted in China [142]. After pyrolysis process, all residues (biochar, bio-oil and syngas) were found to contain HMs. HMs transportation via different applications of pyrolysis byproducts was feasible [106]. Low environmental risk for resource use was achieved by coupling pyrolysis with acid washing (possible ecological risk index values were less than 20) [143]. In their study, they reported the ideal washing conditions by H_3_PO_4_ (acid content of 2.47 mol/L, L/S of 9.85 mL/g, and a washing temperature of 71.18 °C), the overall maximum HM removal efficiency was 95.05%. From the perspective of using solid waste, this work offers pyrolysis along with acid washing treatment for SS as an environmentally favorable alternative.

#### 3.2.9 Wet oxidation

**Fig. 4:**
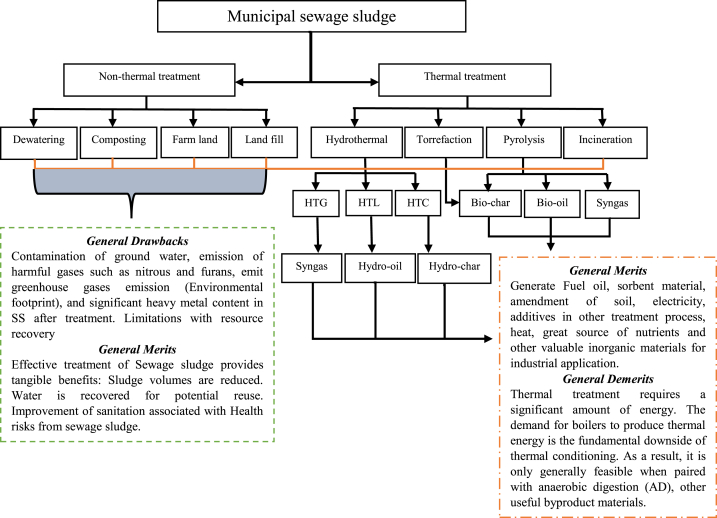
Schematic diagram of thermal and non-thermal treatment of sewage sludge.

Wet oxidation is hydrothermal process through which inorganic and organic material are oxidized at certain temperature and pressure range about (150–360 °C) and (30–250 bar) respectively, plus by maintaining high pressure in liquid phase, leads to the enhancement of oxidation degree [144]. This is complex process and most of the time classified into two major stages such as (a) mass transfer process of oxygen from the gas phase to the liquid phase; (b) Chemical reaction between dissolved oxygen and matrix [145]. Effluent from wet oxidation treatment is carbon-rich material, which can be used in denitrification instead of methanol [146]. In addition, this method considered as the new alternative technology to incineration process, because its furans, dioxin, sulphur dioxide and nitrogen oxides free emissions. Currently, around 200 wet oxidation industries are constructed for treating sludge, chemical, pharmaceutical and petrochemical plants [147]. Although, this thermal treatment doesn't emit some harmful gas into surroundings, it was still inadequate for treating SS. Due to the thermal treatment partial carbon conversion, it allows the capture of toxic HMs (Nickel and vanadium) in the effluent [148]. In addition, Fig. 4 present different treatment routes of MSS via non-thermal and thermal methods.

As it can be observed from Fig. 4, non-thermal technique shows severe contamination than thermal treatment process. Although, some thermal technology such as hydrothermal (gasification, liquefaction and carbonization), torrefaction and pyrolysis has notable environmental and economic benefits, in terms of bio-energy generation and nutrient recovery, which can be useful in many applications for example, in agriculture and other industrial purpose. Some studies reported that, before byproducts from those thermal processes can be applied in any field, upgrading is strongly recommended to avoid second pollution, due to the high concentration of HMs in SS. Non-thermal approaches were found inadequate for treating SS in terms of bioenergy production. In addition, Table 3 compares different treatments technologies of SS with their operating parameters, benefits and drawbacks.

**Table 3:** Thermal and non-thermal treatment method of sewage sludge.

Treatment	Parameter	Principles	Drawbacks	Benefits	Reference
Landfilling	–	A bottom liner, a leachate collection system, a cover, and the natural hydrogeologic setting.	Generation of leachate, waste of valuable organic/inorganic materials, emission of greenhouse gases if not engineered	Cheap process with no energy consumption, reduces SS volume	[85,87]
Farm land	–	Direct use of SS without any treatment	Contamination of heavy metals	Great source of inorganic/organic substances as fertilizer or to improve soil properties	[81,149]
Drying	60–93 °C.	Spread of waste/SS on open bed of sand and allowed to remain until dry	Climate dependent, unstable temperature conditions, cover large land surface, emit odor, heavy metals pollution	Performed in natural way, affordable, destroy pathogen, reduce sludge volume	[136,138]
Dewatering	30–70 °C and 1–300 bar	Minimization of water content from SS for its effective disposal	This treatment process can't mitigate HMs pollution in SS	Improve bio-energy utilization, reduce volume, easy transportation, lessen leachate in landfills, does not contribute to carbon emission	[61]
Torrefaction	>300 °C	Low/slow thermal process in oxygen starving environment	Pollution of HMs through different application of torrefied product	Produce high amount of biochar, improves solid fuel properties, low energy consumption, short torrefaction time, enhance carbon fixation and hydrophobicity of torrefied material	[134,135]
Incineration	Temperatures of 750–925 °C (1400–1700 °F). Residence times are typically 2–5 s.	Straight combustion in excess oxygen supply	Expensive, emit some pollutant gases such us furans and nitrous, ammonia emission, leads to the second pollution via residue (ash)	Odor minimization, reduction of raw waste mass (SS), production of energy.	[116,119]
Composting	55–65 °C	Utilization of microorganism in decomposition of organic material	Contamination of heavy metals, emit harmful gases such as greenhouse gas which leads to the global warming effect.	Biological degradation, generate macro/micro nutrients, destroy pathogens, compost can be used in soil amendment	[26,74]
Pyrolysis	400–800 °C and 2–9 bar	Thermal degradation without any supply of oxygen	Pollution of heavy metals via different application of its byproducts	Generate bio-oil, biochar and syngas which can be used as additives in other treatment process and good source of electricity and fuel	[106,139]
Wet Oxidation	150–360 °C and 30–250 bar	Oxidizing SS in liquid phase with dissolved oxygen at high temperature	Partial convention of carbon allows the capture of heavy metals in effluent	Free emission of harmful gases such as nitrous and furans, produce carbon rich-materials.	[146,148]
Hydrothermal carbonization	180–260 °C and 20–100 bar	Thermal process by converting organic waste/biomass into carbon-rich material	Pollution of toxic heavy metals via different application of it by product	Generate carbon rich material (Biochar), it influence dewaterability, hydrophobic, it is great source of solid fuel	[107,111]
Hydrothermal liquefaction	280°C-370 °C and 100–250 bar	Thermochemical conversion of biomass into hydro-oil	Contamination of polycyclic aromatic hydrocarbon compounds and HMs	Produce high amount of hydro-oil which can be used as fuel.	[113,114]
Hydrothermal gasification	360–700 °C and 210–300 bar	Thermal process for biomass decomposition in inadequate oxygen condition to produce syngas	Syngas produced possibly contains toxic heavy metals.	Allows recovery of chemicals and bio-energy, great source of quality/quantity syngas.	[101,150]
Stabilization	–	Operated through aerobic, AD, composting and vermistabilization	It can be stressed with many factors such as phytochemical properties of waste, pH, temperature, pressure, time etc …	It reduce pathogen, eliminate offensive odor, improve aesthetic and minimize the potential of putrification	[78,80]

## 4 Combined process technology with resource recovery

### 4.1 Co-process integration of plasma pyrolysis and anaerobic digestion

Both plasma pyrolysis and AD have their own advantages and disadvantages, and the choice between the two depends on the specific needs of the sewage treatment facility. Plasma pyrolysis is more suited for facilities that need to dispose of large volumes of SS and produce syngas, Char and allow adsorption of MHs while AD is more suited for facilities that have a consistent supply of SS and are looking to generate energy and digestate. Moreover, integrating plasma pyrolysis with AD treatment process has great potential in terms of energy production, chemical agents and organic nutrient recovery to be applied in different fields. Both processes are considered as thermal technology which operated without supply of oxygen, however plasma pyrolysis performed at high temperature compared to the AD technique. Any type of waste (organic/inorganic materials) can be treated with plasma pyrolysis while AD treats only the organic wastes but the two methods are well known to take full advantage of the wastes in environmental friendly manner. Additionally, byproducts from those treatment methods are all essential materials since they can be used in production of heat, electricity, bio-fuel and nutrient for growing crops. Further, this will come as solution to solve environmental problems caused by the use of nonrenewable energy resource such as fossil fuel.

**Fig. 5:**
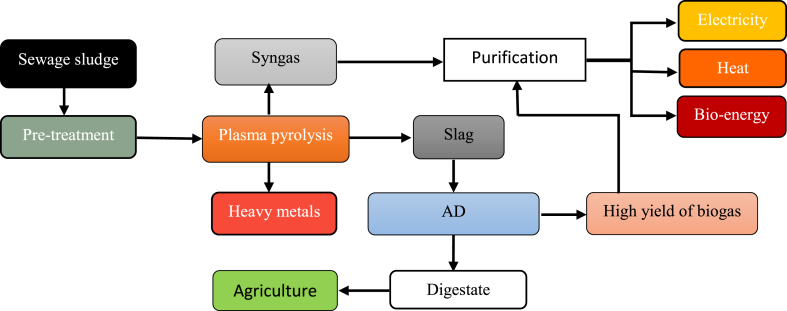
Proposed schematic diagram integration of plasma pyrolysis and Anaerobic digestion.

SS as waste is saturated with carbon, pathogens HMs and other inorganic substances [28]. This makes sludge fit for this co-process for improving and utilize all materials that it contains. Although, AD is reported to treat SS in very economic and ecological friend as it can promote reduction of chemical oxygen demand, nitrogen recycling, recover carbon by biogas production, MHs stabilization and downgrade pathogen. Direct feed of SS into AD treatment process isn't recommended, due to the accumulation of HMs in digestate at the end of treatment. Toxic HMs can inhibits production of biogas and stability of the process [97]. Therefore, to enhance the production of bio-energy from this process and lower chance of second pollution, SS should feed in plasma pyrolysis before, hence the full treatment of SS through syngas, adsorption of MHs and Char generation. Byproducts from this process should be advanced and polished for further application, while Char could be used as additive in AD treatment to influence biogas production and stabilize the process, and also increase amount of nutrients in digestate to be used in field of agriculture. Moreover, diagram of co-process integration of plasma pyrolysis and AD is well shown in Fig. 5.

As presented in Fig. 5, the SS before conveyance into the plasma pyrolysis reactor have to be pre-treated by some method such as dewatering process, afterwards sludge can be heated in plasma pyrolysis to produce syngas. In fact this process takes place at extremely high temperature which leads to the vaporization of MHs from raw SS, and before it feeds to the plasma pyrolysis, present HMs have to be well identified, as SS varies different characteristics day by day. Therefore HMs can be adsorbed at certain temperature based on their difference in vaporization temperature respectively. This will not only help in syngas refining but also prevent the accumulation of HMs in Char at the end of the process. Through this method we can generate free HMs syngas and Char. And also this could be beneficial and great source of HMs to be used in different industrial applications. In addition syngas produced can be polished to generate electricity, bio-fuel or heat. Also it can be great source of H_2_, which can be employed in many industrial sectors as green fuel [130]. In past years, Char produced from plasma pyrolysis was used in construction practices, which can lead to the waste of valuable resources such as inorganic substances and possibly can cause second pollution. To overcome this problem, Han et al. (2019) [131] described Char outstanding and effective additive in AD process, in terms of process stabilization by reducing inhibitors such as ammonia nitrogen, modulate pH, reduce chemical oxygen demand of the raw organic materials and also it increase amount of nutrients in digestate. Hence, high production of quantity/quality of biogas to be purified to generate bio-fuel, heat and electricity and also nutrient saturated digestate can be collected at the end of the process to be applied in agriculture area as organic fertilizer.

### 4.2 Techno-economics of co-plasma pyrolysis and anaerobic digestion

Plasma pyrolysis and AD are both methods of SS treatment that aim to reduce the volume of sludge and stabilize its organic content. In current review, we introduced sustainable and environmental friendly treatment of MSS. And also by comparing co-treatment of plasma pyrolysis and AD process with other treatment methods such as drying, incineration, stabilization, wet-oxidation, composting, gasification, carbonization, pyrolysis, farm land etc … the SWOT (strength, weakness, opportunity and threats) analysis showed that, our suggested technological route has optimal treatment advantage in terms of bio-energy production, adsorption of HMs and nutrient recovery [123]. Although, SS is described as harmful waste as carbonic material that contain pathogens, MHs and other inorganic substances. On the contrary, it makes SS best and renewable raw-feed to this proposed method. In addition, this route leads to the high quantity/quality of syngas production from decomposition of SS in plasma pyrolysis reactor. Syngas generated, via purification process it can be great source of chemicals agents such as H_2_, bio-fuel and electricity, which creates three different valued-added products with different market targets.

Temperature is essential factor in adsorption process and plasma pyrolysis process can promote MHs adsorption in gaseous state as performed at extremely high temperature in absence of oxygen. Li et al. (2013) [151] showed that, by the increase of temperature the discharge rate of MHs also increased and the sequence depends on each and every metals vaporization degree, where rate of discharge is followed in the series Zn > Cu > Pb > Cr > Cd. Moreover, MHs can be adsorbed by thermal process without any use of chemical reagent for solving financial and environmental issue. Ji et al. (2021) [152] synthesized a new technology of thermal responsive absorbent, A-MIL-121, that can adsorb MHs such as Cu (II) (>95%), when the temperature raised leads to the increase in its adsorption capacity towards some other metals such as Cd^2 +^, Ni ^2+^, Pb^2+^. This post SS as renewable source of HMs and solve economic and eco-system problems, due to that MSS known as waste which contains high amount of MHs. Thus, by plasma pyrolysis generate another value product which can be used in different industrial applications.

Moreover, char produced from plasma pyrolysis has much potential to be used in agriculture sector. As it was reported as a suitable source of inorganic materials for instance potassium, phosphorous and nitrogen, which can serve as fertilizer for growing crops. Biochar applications have many environmental benefits. Biochar must be applied in large amounts for worldwide benefits, though it's expensive and difficult to activate in some cases, limiting its practical application. These challenges raise production costs, making many biochar applications unsustainable. In seeking a sustainable alternative, biochar produced from biowaste digestate using waste heat from methane combustion at a biogas plant has various synergies that allow for low production costs and year-round quality [153]. For the first time, techno-economic considerations suggest using this competitive advantage to prevent mass-scale eutrophication. Further, the evaluation includes a suggestion for digestate biochar activation and wastewater treatment plant utilization and a financial estimate of the new application's profitability. However, in the context of the current rising economic crisis, it is critical to compare results with previous research and comprehend some insights linked to bankruptcy risk and profits manipulation, such as debt covenant violations, capital concerns, and the company's financial health [153].

Some studies showed char as best additive to enhance and stabilize some other treatment process such as AD [103]. The char equally improves biogas produced from AD reactor which consist of methane and carbon dioxide; this can be polished and employed as electricity, heat and energy generator. Char from the hazardous waste, such as MSS is another added value byproduct production that could be commercialized, used to promote nitrogen in soil, carbon reutilization, and decrease emission of greenhouse gas in the atmosphere by promoting green treatment process. Char increases inorganic nutrients in AD digestate (after treatment process) to be applied in farming process for soil amendment. However, around 60% of organic materials are degraded in AD treatment, the digestate can further be decomposed in plasma pyrolysis to promote utilization and optimum generation of bioenergy. Additionally, all of those essential byproducts will be selling at affordable price based on economic evaluations and offers zero waste from SS management and indorses policy of circular economy. The amount of money that can be made from plasma pyrolysis coupled with AD of SS will depend on several factors, such as the size and capacity of the treatment facility, the cost of electricity and other resources, the cost of waste disposal or treatment, and the demand for the products produced by these technologies (such as syngas/hydrogen, biogas/methane gas, Char or HMs for other industrial applications). In general, plasma pyrolysis and AD can provide cost savings compared to traditional methods of waste disposal and energy generation. The energy generated from these processes can be used to power the treatment facility or sold to the grid, providing an additional source of revenue. In addition, the solid and liquid residues produced by plasma pyrolysis can be further processed into value-added products, such as biochar, which can be sold as an inorganic fertilizer or soil conditioner. However, it is important to note that the financial viability of these technologies will vary depending on the specific circumstances and location of the treatment facility.

Due to the high temperature expended during plasma pyrolysis, it was viewed as costly and energy-demanding, but the cost can be offset by the quality of energy byproducts yielded during operations. According to Tang et al. (2010) [154], in their preliminary investigation, the average gas yield was 66% and the average syngas concentration was 76%. For a typical capacity of 1800 tons/year with a utilization rate of 0.68 (6000 h/year), the total electric power of plasma pyrolysis installation will be 240 KW, which can process biomass feedstock at 300 kg/h and cost 1200 tons of syngas per year. Installing the plant costs $1.5 million, and repaying it over 15 years costs $300,000. Biomass costs $28 per ton and electricity 0.05 per kWh. Syngas costs $354 per ton with 1500 tons of annual production. They further elucidated that the over-cost of electric power consumption was more than counterbalanced by the production of high concentrations of H_2_ and CO, byproducts like low-cost activated carbon or a semi-reinforcing carbon black precursor, feedstock (due to the theoretical 100% carbon yield), CO_2_ removal, and NO_x_ and SO_2_ reduction. This investigation presented the effectiveness of plasma pyrolysis, which can equally be channeled towards the valorization of SS to provide a means for syngas production in large industrial waste water resource recovery facility conversion plants, even though this was a preliminary examination of the costs of plasma pyrolysis of biomass. Thus, detailed economic analysis of the costs and potential revenue streams would be necessary to determine the exact amount of money that can be made from plasma pyrolysis coupled with AD for SS in a particular setting.

## 5 Challenges and recommendations

Many of researchers has conducted different technological treatment methods to reduce toxicity and pollution of MSS in order to generate bio-energy, adsorption of HMs and nutrient recovery. Here is a perspective and future outlook for both methods: plasma pyrolysis is a relatively new technology that uses high-temperature plasma to break down organic matter in SS. The process is highly efficient and can reduce the volume of sludge by up to 90%, while also producing syngas and a solid residue that can be used as fertilizer or additive in other treatment process. The main advantage of plasma pyrolysis is that it can treat sludge with high moisture content, which is typically a problem for other thermal treatment technologies. However, the technology is still in the early stages of development and has not yet been widely adopted. Future research will focus on enhancing the efficiency of the process, reducing operating costs, and developing applications for the byproducts. The following elements can be used to identify the techno-economic factors for profit analysis: the net energy output, the sales of solid residue, and the gate/tipping fee, by taking into account power consumption and generation, net energy output may be estimated. A steam boiler, steam turbine, or reciprocating gas engine can be used to generate power. The cost of power generation is also heavily influenced by its efficiency [155]. Carbonaceous residue and/or slag formed from the inorganic component of waste feed comprise the solid residue from plasma pyrolysis. Both sorts of processed residue can be sold. Depending on its composition and market demand, the projected general range for solid residue is from $0 to $25 per ton [156].

Additional challenges to be considered are the operating parameters of plasma pyrolysis with SS waste should be addressed to improve/optimize syngas generation and HMs adsorption. Due to, temperature and residence time are key factors, which has an effect on generated syngas properties, vaporization of HMs and Char characteristics. Treatment process conditions for increasing production of hydrogen gas and reduce harmful elements in syngas have to be investigated. Although, high temperature operational condition could induce inertization process of the HMs in the SS valorization, but the HMs transportation via different applications of pyrolysis byproducts are still feasible [106]. The HMs can be significantly reduced with combination of plasma pyrolysis with chemical process such as acid washing with H_2_PO_4_ [143]. As reported by some studies, during plasma pyrolysis process some small amount of harmful gas can be emitted and also owing to the process parameters can increase or decrease hydrogen in syngas produced. Additional study is needed to advance absorption of HMs and set perfect temperature and time for this process.

It is well known that metals have different vaporization temperature and time, hence setting perfect temperature for respective metals is essential factor, which will help in high amount adsorption of HMs and influence the production of clean syngas and Char. To diminish haz-ardous elements in the syngas, alkali based wet scrubbers and other filters may be used at the downstream of the plasma pyrolysis system [155]. The construction of a small, cost-effective reactor that will attract the private sector; Use of graphite electrodes as opposed to metal electrodes to prevent corrosion; Modeling and simulation of thermodynamics for process implementation, as well as a comprehensive energy and economic analysis for cost optimization were recommended for plasma pyrolysis commercial application [123]. The Char is highly affected with temperature of the process, as SS is considered as puzzle waste, it may contain some materials which can inhibits AD process or be accumulated in soil which might have negative impact in long term period. The efficacy of biochar in AD was reported to promote high methane yield and reactor stability within 350 days at higher OLR of 6.0 gVS/L^-d^, digestate quality desirable for agriculture has improved with higher concentrations of micro and macronutrients constituents compared to the control, but caution and further investigation on the appropriate quantity of biochar insertion into AD process have to be examined and the raw substrate quality for biochar production to avert AD inhibition [157].

AD is a well-established technology for SS treatment that has been used for decades. The process involves the decomposition of organic matter by bacteria in the absence of oxygen, which produces biogas and a nutrient-rich liquid residue (digestate), and the digestate liquor can be utilized to replace freshwater and nutrients in algae cultivation [158]. However, the AD process is limited by the high water content and presence of HMs in SS, which can lead to reduce the efficiency of digestion. Studies are highly recommended to improve the efficiency by reducing inhibitors and stabilizing the process, exploring new applications for the byproducts, and advancing technologies to overcome the challenges of MHs and high moisture content of sludge towards a circular economy. Because of the need for efficient resource use and waste management, the transition to a circular economy is considered a necessity; thus, resource efficiency is becoming more critical for entrepreneurs, and they are looking at circular business models to turn their waste into assets [159]. Looking to the future, it is likely that plasma pyrolysis and AD will continue to be used for SS treatment. There will likely be an increased focus on improving the efficiency and sustainability of these processes towards a sustainable circular economy. Efforts may be made to reduce the energy required for plasma pyrolysis, increase quality/quantity of its byproducts and to advance the efficiency of biogas production in AD process. Additionally, there may be a greater emphasis on using the byproducts of these processes for other applications. For example, the solid residue produced by plasma pyrolysis can be used as a feedstock for the production of biochar and also other different applications of their value-added products must be investigated.

## 6 Conclusion

The AD of the biodegradable fraction of SS is a common technique for producing biogas and digestate for agronomy. However, the biodegradable component of SS contains HMs, which impairs digestate production quality. As a result, the SS must first be transferred for alternative downstream treatment by plasma pyrolysis in order to yield valuable compounds. This plasma pyrolysis produces waste-to-value-added products such as syngas, hydrogen, and char, resulting in significant bulk and volume reduction. Furthermore, it conserves a large amount of land, which is required for other approaches such as landfills. Power supply, reactor temperature, feed rate, feed composition, and electrode spacing all have an impact on process output. Plasma pyrolysis consumes a lot of energy, especially when it comes to capital and maintenance costs. On the other hand, the cost of operating high-temperature plasma can be repaid through the generation of syngas, hydrogen, and char, making the technique cost-effective. Char includes a high concentration of important elements such as nitrogen, phosphorus, and potassium and can be used as an organic fertilizer. This substance could also be used as an adsorbent and an addition to the AD bioprocess in other treatment procedures. The addition of Char to the upstream AD process increases the quantity and quality of biogas produced while also enhancing the nutrients in the digestate. The AD can further transform the organic material into biogas, a renewable energy source, and a nutrient-rich residue that can be used as fertilizer, while minimizing the volume of SS material produced. The combination of these two technologies has the potential to improve efficiency, sustainability, and the creation of new applications for byproducts in the future. The integration of plasma pyrolysis and AD may provide an environmentally benign and cost-effective alternative for SS management.

## Author contributions

All authors listed have significantly contributed to the development and the writing of this article.

## Funding

This work was supported by the start-up Funding for Research of Nanchang Institute of Science and Technology (NGRCZX-22-03), School of Environment and Civil Engineering, Nanchang, Jiangxi-China. Nanchang Key Laboratory (project number: 2021-NCZDSY-007), and the Jiangxi Province Key R&D Program General Project:20202BBGL73084.

## Data availability statement

Data will be made available on request.

## Declaration of interest's statement

The authors declare no conflict of interest.

## Additional information

No additional information is available for this paper.

## Declaration of competing interest

The authors declare that they have no known competing financial interests or personal relationships that could have appeared to influence the work reported in this paper.
